# Surgical Induction of Mid‐Thoracic Aortic Coarctation in Mice: A Reproducible Preclinical Model of Pressure‐Induced Vascular Remodeling

**DOI:** 10.1002/cpz1.70318

**Published:** 2026-02-16

**Authors:** D. Adam Lauver, Hannah Garver, Teresa Kreiger‐Burke, C. Javier Rendón, G. Andres Contreras, Stephanie W. Watts, Gregory D. Fink

**Affiliations:** ^1^ Department of Pharmacology and Toxicology Michigan State University East Lansing Michigan USA; ^2^ Department of Large Animal Clinical Sciences Michigan State University East Lansing Michigan USA

**Keywords:** arterial pressure gradient, cardiovascular physiology, hemodynamics, hypertension, mouse model

## Abstract

Elevated arterial pressure is a key contributor to cardiovascular disease, yet experimental models that isolate the effects of pressure from confounding systemic factors remain limited. We describe a reproducible surgical protocol to induce mid‐thoracic aortic coarctation in mice, generating a stable and quantifiable arterial pressure gradient within the same animal. Using a biocompatible rubber O‐ring, a partial stenosis is applied to the descending thoracic aorta of anesthetized C57BL/6J mice via thoracotomy. The resulting model establishes upstream hypertension while preserving distal perfusion, enabling the investigation of pressure‐specific effects on vascular structure and perivascular adipose tissue function. Hemodynamic assessment by radio telemetry and high‐frequency Doppler ultrasound confirms significant and sustained gradients in mean and systolic blood pressure across the coarctation site. This model provides a valuable tool for studying pressure‐induced remodeling and vascular biology in a controlled and physiologically relevant context. © 2026 The Author(s). *Current Protocols* published by Wiley Periodicals LLC.

**Basic Protocol 1**: Induction of mid‐thoracic aortic coarctation using a rubber O‐ring

**Basic Protocol 2**: Measurement of arterial pressure gradient using two PA‐C10 radio telemeters

**Basic Protocol 3**: Measurement of blood flow velocity in the descending thoracic aorta using doppler ultrasound

## INTRODUCTION

Arterial blood pressure is a fundamental determinant of cardiovascular structure and function. Chronic elevations in pressure contribute to a range of pathologies, including vascular remodeling, atherosclerosis, and heart failure (Harrison et al., 2021). Understanding the precise impact of blood pressure on vascular and perivascular tissues, such as perivascular adipose tissue (PVAT), is essential for dissecting mechanisms of cardiovascular disease progression (Queiroz & Sena, 2024; Watts et al., 2020). However, many experimental models that elevate blood pressure, such as chronic angiotensin II infusion or renal artery stenosis, introduce systemic changes beyond pressure elevation alone, complicating mechanistic interpretation (Coffman, 2014; Lerman et al., 2019; Rodriguez‐Iturbe et al., 2017).

To address this limitation, we present a murine model of mid‐thoracic aortic coarctation that establishes a controlled and sustained pressure gradient along the descending thoracic aorta. This technique enables researchers to investigate pressure‐specific effects in upstream vs downstream vascular segments within the same animal, thereby eliminating systemic confounders. The procedure involves the production a partial constriction to the mid‐thoracic aorta via thoracotomy, using a biocompatible rubber O‐ring. This procedure generates a reproducible and tunable narrowing that results in elevated pressure proximal to the coarctation while preserving distal perfusion.

This model permits precise measurement of arterial pressure gradients via radio telemetry, as well as assessment of local hemodynamics using pulsed‐wave Doppler ultrasound. The resulting data include real‐time pressure recordings from carotid and femoral arteries, heart rate, pulse pressure, and peak blood flow velocities across the coarctation site. These measurements can be correlated with molecular and histologic changes in vascular or PVAT tissues, enabling detailed investigations of mechanotransduction, inflammation, and tissue remodeling.

Compared to traditional models of systemic hypertension, the mid‐thoracic coarctation model offers the major advantage of spatial specificity since pressure is elevated in only part of the vasculature. This feature enables internal controls and provides more direct attribution of observed effects to mechanical forces. The disadvantages include the need for surgical expertise and specialized equipment for telemetry and imaging, as well as the inability to model systemic hypertensive pathophysiology. The technique has evolved from earlier transverse aortic constriction (TAC) models, with adaptations here enabling chronic pressure differentials suitable for long‐term studies of vascular biology.

This article includes three protocols designed to establish and assess a mid‐thoracic aortic coarctation model in mice. Basic Protocol 1 describes the use of a nitrile rubber O‐ring to create a standardized constriction of the thoracic aorta, minimizing operator variability and ensuring reproducibility. Basic Protocol 2 describes the use of two independent radio telemetry devices to simultaneously record arterial blood pressure from the carotid and femoral arteries in anesthetized mice, enabling direct calculation of the pressure gradient across the aortic coarctation. Basic Protocol 3 outlines the use of high‐frequency Doppler ultrasound (e.g., Vevo F2) to non‐invasively measure blood flow velocity in the descending thoracic aorta, providing spatially resolved confirmation of flow disturbance and vessel narrowing.

*NOTE*: All procedures described in these protocols have been reviewed and approved by the Michigan State University Institutional Animal Care and Use Committee (IACUC) and have been conducted in accordance with the Guide for the Care and Use of Laboratory Animals and applicable AVMA guidelines. The proposed studies are covered under MSU IACUC Protocol #PROTO202400255.

## Basic Protocol 1 INDUCTION OF MID‐THORACIC AORTIC COARCTATION USING A RUBBER O‐RING

**Figure 1:**
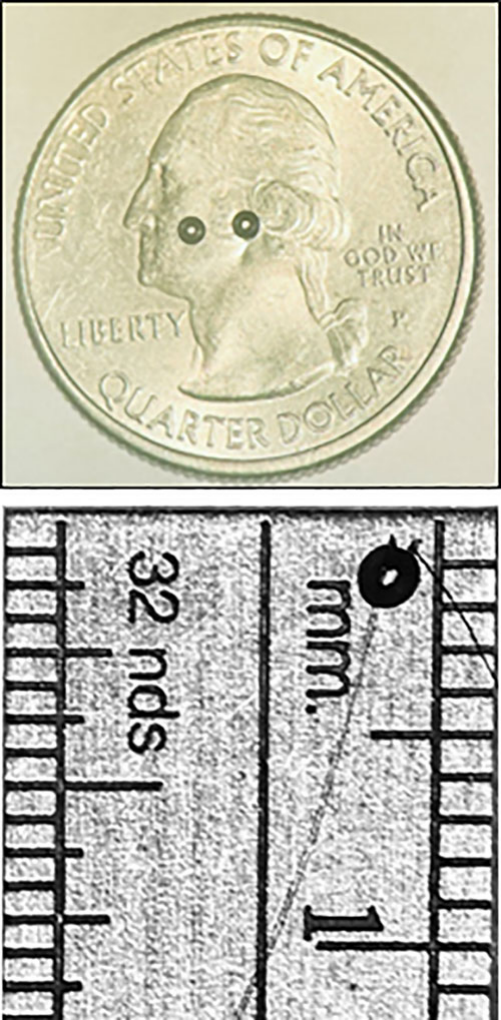
A stable pressure gradient is established in the thoracic aorta of mice by applying a rubber (nitrile) O‐ring, pictured with a quarter and ruler for scale.

This protocol describes the surgical induction of mid‐thoracic aortic coarctation in mice using a biocompatible rubber O‐ring (Fig. 1). The goal is to create a partial and stable constriction of the descending thoracic aorta that generates a reproducible arterial pressure gradient between the proximal and distal vasculature. Mice are anesthetized with isoflurane, intubated, and mechanically ventilated. A left thoracotomy is performed to expose the aorta, and a nitrile O‐ring is placed around the vessel without disrupting surrounding tissues. The size and elasticity of the O‐ring are chosen to allow blood flow while producing measurable hemodynamic changes.

This method, adapted from Nakao et al. (2022), offers a standardized and low‐variability approach to coarctation, ideal for laboratories seeking reproducibility across experiments or operators. When performed correctly, mice should recover uneventfully and develop a stable pressure gradient (typically 10 to 15 mmHg) between the carotid and femoral arteries. The model supports long‐term studies of pressure‐induced vascular remodeling, perivascular adipose tissue responses, and other downstream physiological changes (Contreras et al., 2020).

**Figure 2:**
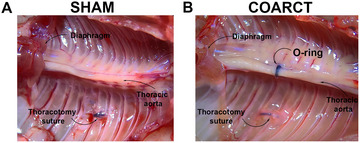
Representative surgical images from (**A**) a sham animal and (**B**) an animal in which a nitrile O‐ring had been placed around the thoracic aorta. The coarctation was placed via a thoracotomy, which was performed between the fourth and fifth intercostal spaces. Experiments were performed in adult mice anesthetized with isoflurane (2% in oxygen) and artificially ventilated. Images were taken at the end of the 8‐week recovery period.

The placement of the O‐ring for mid‐thoracic aortic coarctation is guided by both anatomical landmarks and physiological considerations to ensure consistent and reproducible pressure gradients across animals. The O‐ring is typically positioned just distal to the left subclavian artery, along a segment of the descending thoracic aorta that is accessible via left thoracotomy and free of major arterial branches (Fig. 2). Several factors may influence the precise placement, including mouse size, age, strain, and sex, as these can affect thoracic cavity dimensions, aortic compliance, and vessel orientation. For example, younger or smaller mice may have shorter or more delicate aortic segments, requiring careful dissection to avoid injury. Strain‐dependent differences in aortic anatomy, such as the branching pattern of intercostal arteries or vessel thickness, can also impact the ease and accuracy of O‐ring positioning. While the target region remains consistent across experiments, the surgeon may need to make minor adjustments based on real‐time visual assessment of aortic exposure and anatomy to ensure proper constriction without compromising distal perfusion.

### Materials

C57BL/6J mice, 20 to 30 g, 6 to 8 weeks of age (The Jackson Laboratory, strain no. 000664)
Surgical anesthetics/analgesics:
  Isoflurane (Piramal, NDC# 66794‐017‐25)
  Buprenorphine (Ethiqa XR, Fidelis Animal Health, NDC# 86084‐100‐30)
  Enrofloxacin (Enroflox, Norbrook Laboratories, NDC# 55529‐154‐01)
  Carprofen (generic, Dechra Veterinary Products, NDC# 17033‐351‐18)
  Optixcare ophthalmic lubricant (Aventix Animal Health Corp, OPX‐4242)

Anesthesia unit, i.e., vaporizer calibrated for isoflurane use, induction box, nosecone circuit (E‐Z Systems, EZ‐SA800)
Benchtop anesthesia scavenging system (Sentry Air Systems, SS‐200‐WSL)
Endotracheal intubation kit (Kent Scientific, ETI‐MSE)
Intubation stand (Kent Scientific, ETI‐MSE‐01)
RoVent Jr. small animal ventilator (Kent Scientific, RV‐JR)
Surgical stereoscope (Leica Wild M695 or similar)
Surgical supplies:
  Chemical depilatory (e.g., Nair for sensitive skin, 049‐06‐1388)
  0.3‐ml BD insulin syringes (Medline, BD‐324910Z)
  1.0‐ml BD insulin syringes with 25G needle (Medline, BD‐303060)
  InSyte Autoguard shielded IV catheter, 22G, for use as endotracheal tube (Becton Dickinson, 381423)
  6‐0 silk suture (Ethicon, Med Vet International, 786G)
  8‐0 nylon suture (Fine Science Tools,18030‐80)
  Microneedle holder (Fine Science Tools, 12075‐12)
  Dumont forceps (Fine Science Tools, 11203‐23)
  Semken forceps (Fine Science Tools, 11024‐12)
  Moria forceps (Fine Science Tools, 11370‐40)
  Vannas spring scissors (Fine Science Tools, 15044‐08)
  Metzenbaum mini scissors (Fine Science Tools, 14074‐09)
  Olsen‐Hegar needle holder (Fine Science Tools, 12002‐12)
  Alm retractor (Fine Science Tools, 17008‐07)
  O‐rings, 0.017 × 0.020–in. 50 BUNA‐N, nitrile (Apple Rubber Products, R00017‐020‐50BNB)
  Surgical closure AutoClips, 9‐mm (Texas Scientific Instruments, 205016)
  0.9% sodium chloride injection, USP (Dechra Veterinary Product, NDC# 17033‐502‐01)
Heating pad and circulating water pump (Jorgensen Labs, J0783X)
Small animal heat lamp, infrared bulb (Physitemp, HL1)

*NOTE*: All surgical steps listed below are performed identically in sham‐operated animals (sham controls), with the exception that no O‐ring is placed around the aorta. Sham procedures are essential to control for surgical and anesthetic effects unrelated to aortic constriction.

#### General anesthesia induction and maintenance

The mouse will be placed in an induction chamber and anesthetized using 2.0% to 4.0% isoflurane delivered in 1 L/min of oxygen. Once anesthetized, the animal will be maintained under surgical anesthesia with 1.0% to 2.0% isoflurane in 1 L/min of oxygen. The mouse will then be intubated and mechanically ventilated, with ventilator settings automatically adjusted based on the animal's body weight (in grams). The depth of anesthesia will be continuously monitored by assessing respiratory rate, absence of spontaneous movement, and lack of response to a brief tail pinch using forceps.

Prior to surgery, mice will receive preoperative antibiotics [enrofloxacin, 5 mg/kg subcutaneously (SC)] and analgesics (buprenorphine, 3.25 mg/kg SC, and carprofen, 5 mg/kg SC) to prevent infection and minimize pain. Ophthalmic ointment will be applied to both eyes to prevent corneal drying during the procedure.

#### Surgical procedure

1 Perform a left thoracotomy at the 2nd to 3rd intercostal space using sterile technique to access the thoracic cavity.
  This location provides optimal access to the descending thoracic aorta while minimizing disruption to surrounding structures.
2 Insert small retractors to gently separate the ribs and expose the descending thoracic aorta.
  Adequate visualization of the aorta is critical for precise placement of the constriction device.
3 Using a surgical stereoscope, carefully dissect a short segment of the aorta free of surrounding tissue, then place a pre‐measured nitrile O‐ring around the aorta and suture its ends together to complete the ring.
  The O‐ring must be cleanly pre‐cut before placement, as it cannot be slipped over the aorta without causing significant trauma to the surrounding tissues or the vessel itself. It must be positioned snugly but without compressing the vessel during placement. Closure with suture ensures the ring remains in place without slippage.
4 Place a chest tube into the thoracic cavity to evacuate air and allow for re‐establishment of negative intrathoracic pressure.
  This step is essential to prevent pneumothorax and promote full lung re‐expansion after closure.
5 Close the chest wall in layers using 6‐0 silk suture and withdraw any residual air from the cavity, allowing spontaneous ventilation to resume.
  Proper evacuation of air ensures lung re‐inflation and minimizes respiratory complications post‐operatively.
6 Remove the chest tube and close the skin incision using sterile surgical staples.
  Skin closure should ensure a tight seal to prevent infection and maintain wound integrity during recovery.
7 Keep the animal on the ventilator with oxygen support until it demonstrates clear signs of recovery, including spontaneous breathing, response to toe pinch, and the return of corneal reflexes.
  Continued oxygenation and monitoring during emergence from anesthesia are essential to ensure a smooth and safe recovery. Reflex checks provide reliable indicators of anesthetic depth and neurologic recovery prior to extubation or removal from the heated surgical field.
8 Immediately after surgery, place animals in clean home cages on their side with a warming surface (e.g., circulating water pad or heat lamp) to support thermoregulation during the early recovery period.
  Maintaining body temperature is essential to reduce post‐anesthesia hypothermia and improve survival.

## Basic Protocol 2 MEASUREMENT OF ARTERIAL PRESSURE GRADIENT USING TWO PA‐C10 RADIO TELEMETERS

This protocol describes the use of dual‐channel radio telemetry to measure arterial blood pressure simultaneously in two vascular territories, typically the carotid and femoral arteries, of anesthetized mice. Eight weeks after recovery from aortic coarctation or sham surgery, mice are anesthetized with isoflurane, and pressure‐sensing catheters from a telemetry device (e.g., DSI PhysioTel PA‐C10) are surgically inserted into the target arteries. This setup enables continuous, high‐resolution monitoring of hemodynamic parameters in real time.

The primary objective is to quantify the arterial pressure gradient across the coarctation site with high precision. This approach avoids the confounding effects of restraint or stress and permits direct comparison of upstream (carotid) and downstream (femoral) pressures. In coarcted animals, a stable pressure gradient is typically observed, with mean arterial pressure differences ranging from 10 to 15 mmHg. In addition to pressure gradients, the system allows for concurrent measurement of heart rate, pulse pressure, and other cardiovascular metrics, supporting comprehensive assessment of pressure‐mediated vascular responses over time.

### Materials

Mice (see Basic Protocol 1)
Surgical anesthetics/analgesics:
  Isoflurane (Piramal, NDC# 66794‐017‐25)
  Lidocaine HCl, 2% injectable (Phoenix, NDC# 57319‐533‐05)
Optixcare ophthalmic lubricant (Aventix Animal Health Corp, OPX‐4242)

Anesthesia unit, i.e., vaporizer calibrated for isoflurane use, induction box, nosecone circuit (E‐Z Systems, EZ‐SA800)
Benchtop anesthesia scavenging system (Sentry Air Systems, SS‐200‐WSL)
Heating pad and circulating water pump (Jorgensen Labs, J0783X)
Surgical supplies:
  Dumont forceps (Fine Science Tools, 11203‐23)
  Semken forceps (Fine Science Tools, 11024‐12)
  Moria forceps (Fine Science Tools, 11370‐40)
  Vannas spring scissors (Fine Science Tools, 15044‐08)
  Metzenbaum mini scissors (Fine Science Tools, 14074‐09)
  Alm retractor (Fine Science Tools, 17008‐07)
  1.0‐ml BD insulin syringes with 25G needle (Medline, BD‐303060)
  6‐0 silk suture (Ethicon, Med Vet International, 786G)
Implantable telemeters, TA11PA‐C10 (Data Sciences International)
Telemeter receivers RSC‐1 (Data Sciences International)
Ponemah v6.x software (Data Sciences International)

#### Description of device

The TA11‐PAC10 implantable device is a compact, implantable radio telemetry device designed for continuous monitoring of cardiovascular parameters in mice. The device is cylindrical in shape, weighing ∼1.4 g with a volume of 1.1 ml. Once appropriately instrumented, it enables real‐time acquisition of blood pressure and heart rate data. The device processes these physiological signals and wirelessly transmits them via radiofrequency to a remote receiver connected to a data acquisition system for analysis.

#### General anesthesia induction and maintenance

Anesthesia is induced by placing the mouse in an induction chamber with a continuous flow of 2% to 4% isoflurane in oxygen at 1.0 L/min until a surgical plane of anesthesia is achieved. The animal is then transferred to a heated surgical platform and positioned in dorsal recumbency. Anesthesia is maintained using 2% isoflurane delivered via a nose cone at the same flow rate (1.0 L/min). The depth of anesthesia is continuously monitored throughout the procedure by observing respiratory rate, lack of movement, and the absence of response to tactile stimuli. Ophthalmic ointment is applied to both eyes to prevent corneal drying during anesthesia.

#### Surgical preparation

For femoral artery catheterization, the hair over the ventral abdomen and inner left thigh is shaved to provide a clear surgical field. For carotid artery access, the fur on the neck is carefully shaved. The surgical site is then cleaned and prepped using standard aseptic technique.

#### Surgical procedure for arterial catheterization and pressure gradient measurement

1 Make a small incision to expose the target artery. For femoral artery catheterization, a skin incision is made along the inner left thigh using sharp surgical scissors to expose the femoral neurovascular bundle. For carotid artery access, a midline incision is made over the trachea to expose the left carotid artery.
  Clean and precise dissection is essential to minimize tissue trauma and maintain vessel integrity.
2 Isolate a ∼10‐mm segment of the artery using non‐serrated fine‐tipped forceps.
  Gentle handling reduces the risk of vasospasm and endothelial injury.
3 Pass two pre‐cut 6‐0 braided silk sutures beneath the isolated arterial segment and secure the distal suture with a ligature.
  The distal ligature prevents retrograde bleeding during catheter insertion.
4 Irrigate the exposed artery with 2% lidocaine solution.
  Lidocaine helps to dilate the vessel, reduce vasospasm, and provide local analgesia.
5 Apply gentle upward traction to the proximal suture to temporarily occlude arterial blood flow.
  Temporary occlusion minimizes bleeding during vessel puncture and catheter advancement.
6 Pierce the artery just proximal to the distal tie using a 25G needle with the tip bent at a 90° angle.
  The bent needle facilitates a shallow entry angle into the vessel wall, which is ideal for small‐caliber arteries.
7 Insert the pressure‐sensing catheter into the artery and advance it proximally.
  Catheter advancement should be smooth and gentle to prevent vessel perforation or kinking.
8 Gradually release tension on the proximal suture to allow the catheter to slide into the vessel lumen (advance 2 to 5 cm, depending on anatomy).
  This maneuver uses blood flow to aid catheter advancement while maintaining position control.
9 Secure the catheter in place by tying the proximal suture snugly around both the artery and catheter, followed by the distal tie.
  Both ties are necessary to ensure catheter stability and prevent backflow or dislodgement.
10 Record arterial pressures for 10 to 15 min using the radio telemetry system. Following data acquisition, euthanize the animal according to approved protocols and proceed with tissue collection.
  Ensure data acquisition is free of motion artifacts or signal loss before proceeding to euthanasia and tissue harvest.

## Basic Protocol 3 MEASUREMENT OF BLOOD FLOW VELOCITY IN THE DESCENDING THORACIC AORTA USING DOPPLER ULTRASOUND

This protocol outlines the use of high‐frequency Doppler ultrasound imaging to measure blood flow velocity in the descending thoracic aorta of mice following mid‐thoracic aortic coarctation. The procedure is performed under light isoflurane anesthesia using a high‐resolution imaging platform (e.g., Visualsonics Vevo F2), which allows for real‐time visualization of blood flow patterns both proximal and distal to the coarctation site.

Pulsed‐wave Doppler is used to quantify peak systolic velocities, while color Doppler aids in localizing turbulent flow regions. The velocity ratio between proximal and distal sites serves as a functional indicator of the severity and stability of the coarctation‐induced flow disturbance. When conducted properly, coarcted mice should exhibit a significantly higher proximal‐to‐distal velocity ratio compared to sham‐operated controls. This non‐invasive technique complements pressure telemetry by providing spatially resolved hemodynamic data and can be repeated at multiple timepoints for longitudinal monitoring.

### Materials

Mice (see Basic Protocol 1)
Isoflurane anesthesia (Piramal, NDC# 66794‐017‐25)
Optixcare ophthalmic lubricant (Aventix Animal Health Corp, OPX‐4242)
Chemical depilatory (e.g., Nair for sensitive skin, 049‐06‐1388)
ddH_2_O
Ultrasound gel (e.g., Aquasonic 100, 01‐08, or similar water‐soluble)

Vevo F2 high‐frequency ultrasound (FUJIFILM Visualsonics)
Heated imaging platform containing non‐invasive respiration/ECG monitor (FUJIFILM Visualsonics)
Anesthesia unit, i.e., vaporizer calibrated for isoflurane use, induction box, nosecone circuit (E‐Z Systems, EZ‐SA800)
Benchtop anesthesia scavenging system (Sentry Air Systems, SS‐200‐WSL)
Heating pad and circulating water pump (Jorgensen Labs, J0783X)
Clippers for fine fur (Aesculap Exacta, J1624B, or similar)
Cotton‐tipped swabs (Puritan, 806‐WC)
Cotton gauze (Fisherbrand, 22‐362178)
Paper towels (various)
Adhesive tape (e.g., 3M Transpore, 1527‐0, or similar)
Small animal heat lamp, infrared bulb (Physitemp, HL1)
25 to 57 MHz linear array transducer (FUJIFILM Visualsonics UHF 57×)

1 Turn on the ultrasound unit and imaging platform. Pre‐heat the platform to 37°C.
  The imaging platform contains embedded ECG monitoring strips, respiratory monitoring, temperature control, and input to the ultrasound unit proper.
2 Place the animal into the induction chamber and introduce 3% to 3.5% isoflurane in oxygen at 1 L/min.
3 Remove the animal from the chamber as soon as it becomes immobile and position the animal supine on the heating pad. Maintain anesthesia (1.5% to 2% isoflurane) via use of a nose cone and apply a small amount of eye lubricant onto each eye.
  Anesthetic depth should remain as light as possible throughout the imaging session. Isoflurane is adjusted to maintain immobility of the animal while keeping heart rate >450 bpm.
4 Remove fur over the thorax using clippers, followed by application of depilatory cream using a cotton tip swab.
  Hair should be removed only from the required area. Removing excessive amounts of fur will impact the animal's ability to thermoregulate effectively.
5 Remove cream after ∼30 s by wiping with dry gauze, followed by gauze moistened with water then wiping with a dry paper towel.
  Chemical depilatory cream can be irritating to skin. Exposure time should be minimized, and complete removal/rinsing of the cream is essential.
6 Re‐position animal to sternal recumbency. Remove fur over the left thorax using clippers and depilatory cream as described above.
7 Transfer the animal to the ultrasound imaging platform and position it supine.
8 Apply a small amount of ultrasound gel to each metal ECG surface electrode and carefully tape all four paws in position using gentle adhesive tape.
9 Lubricate the rectal temperature probe with ultrasound gel and carefully insert it into the rectum. Tape the probe and tail to the platform to prevent movement. Monitor temperature and maintain between 36.5° and 37.5°C using a supplemental heat lamp if needed.
10 Place warm ultrasound gel over the sternum region and collect parasternal long‐axis images of heart in 2D B‐mode and short‐axis images in M‐mode at the level of the papillary muscles using a linear array transducer (25 to 57 MHz)
  Record a minimum of two 5 s strips (or 100 frames) of real‐time B‐mode/2D echo from each imaging window for offline analysis. The integrated rail system of the ultrasound unit may be used to stabilize the transducer. Care must be taken to avoid excessive pressure to chest as this may result in bradycardia and/or respiratory compromise.
11 Wipe gel from skin surface using damp (water) gauze, followed by dry gauze or paper towel.
12 Remove tape from paws and temperature probe.
13 Re‐position the animal to a prone position, taking care that the temperature probe does not move excessively within the rectum.
14 Couple paws to ECG surface electrodes using ultrasound gel and gentle adhesive tape.
15 Place warm ultrasound gel over the dorsal thorax, just left of the spine.
16 Using a 25 to 57 MHz linear array transducer, identify the descending thoracic aorta in longitudinal view as well as the location of the arterial constriction using B‐mode and color Doppler.
  A mixed color pattern indicates regions of turbulent or non‐laminar blood flow.
17 Collect pulsed‐wave Doppler images proximal and distal to the coarctation.
  Optimize the color flow and peak velocity signals by aligning the transmitted ultrasound beam parallel to the flow. The angle between the ultrasound beam and flow should be <60°.
18 Clean gel from the animal's skin using damp (water) gauze, followed by gently wiping with a dry paper towel. Remove the temperature probe and remove tape from paws.
19 Transfer the animal into a clean recovery cage above a heating pad set to 37°C.
20 The animal is returned to its home cage upon recovery from anesthesia.
21 The transducer, temperature probe, and imaging platform are cleaned using gauze moistened with ddH_2_O.
22 Measure and evaluate all ultrasound images offline using the ultrasound system's proprietary desktop software (e.g., VevoLab).
  Total anesthesia time, including induction, should not exceed 45 min. Offline analysis assists with workflow and minimizes anesthesia time.

## COMMENTARY

### Critical Parameters

#### Basic Protocol 1: Aortic coarctation surgery

A critical element for success in either coarctation procedure is the clear and atraumatic exposure of the mid‐thoracic aorta. Adequate visualization is essential to ensure accurate placement of the constriction device. Poor exposure can lead to misplaced or uneven constriction, inadvertent vessel injury, or variable and inconsistent pressure gradients.

The selection and consistent use of O‐rings with defined inner and outer diameters (0.017″ and 0.020″, respectively) and appropriate durometer (typically 50 Shore A) is key to reproducibility. Variability in ring stiffness between rubber types may subtly influence the degree of aortic constriction, so the material should be standardized within experiments.

#### Basic Protocol 2: Radio telemetry

In this protocol, proper placement of the pressure‐sensing catheters is vital for accurate measurement of arterial pressure gradients. The catheters must be precisely advanced into the carotid and femoral arteries; misplacement can lead to dampened or artifact‐prone signals that misrepresent true hemodynamics.

Animals should be monitored closely for signs of pain or distress, as inadequate anesthesia can influence cardiovascular parameters and compromise data integrity. Additionally, ensuring proper telemetry calibration and minimizing environmental sources of electromagnetic interference are critical for high‐fidelity signal acquisition.

#### Basic Protocol 3: Doppler ultrasound

Successful application of Doppler ultrasound imaging requires careful alignment of the ultrasound transducer with the aortic axis. Proper positioning is necessary to obtain accurate Doppler angle correction and reliable peak velocity measurements. Even small misalignments can significantly distort velocity readings and impair data quality.

Anesthesia depth must be tightly controlled during imaging. Isoflurane should be administered at the lightest effective dose to minimize physiologic depression without introducing motion artifacts. Finally, probe pressure must be applied gently and consistently. Excessive compression of the thoracic cavity can artificially reduce blood flow velocity and obscure true flow dynamics, particularly in the descending aorta.

### Troubleshooting

See Table 1 for a troubleshooting guide for aortic coarctation, telemetry, and doppler ultrasound.

**Table 1:** Troubleshooting Guide for Aortic Coarctation, Telemetry, and Doppler Ultrasound

Problem	Possible cause	Solution
No pressure gradient observed	Improper placement or sizing of the O‐ring	Recheck aortic constriction and repeat surgery using standardized tools
High mortality after surgery	Excessive constriction or surgical trauma	Minimize manipulation of the aorta
Poor telemetry signal	Misplaced catheter or damaged sensor	Verify catheter placement and test the transmitter
Inconsistent velocity measurements	Poor probe alignment or unstable anesthesia	Adjust probe angle; stabilize anesthesia plane
Low flow velocity in sham animals	Incorrect Doppler gain or probe alignment	Adjust settings and ensure gentle transducer application

### Statistical Analysis

Statistical comparison of blood pressure gradients and velocity ratios between coarcted and sham‐operated animals is best performed using unpaired *t*‐tests or two‐way ANOVA with post hoc corrections where appropriate. Repeated measures ANOVA may be used for longitudinal telemetry data. Power calculations should guide animal numbers to ensure statistical confidence, typically aiming for *n* = 6 to 12 per group.

### Understanding Results

**Figure 3:**
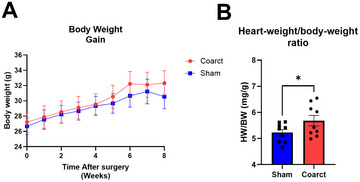
Body weights and heart‐weight/body‐weight ratios after sham or coarctation surgeries. (**A**) Body weights were similar in sham and O‐ring coarcted mice over the duration of the study. (**B**) Heart‐weight/body‐weight ratios were more variable in coarcted animals. The data are presented as the mean ± *SEM*, *n* = 6 to 12 per surgical group.

**Figure 4:**
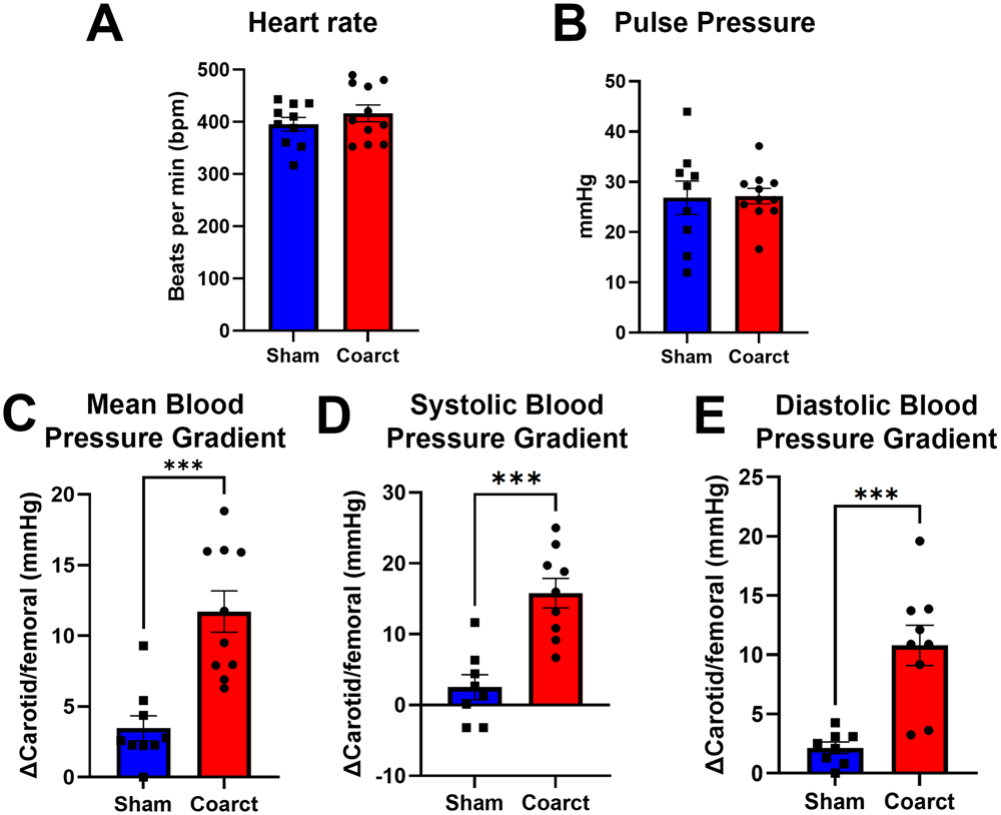
Telemetry data collected from sham or coarcted mice. Heart rate (**A**) and pulse pressure (**B**) were similar in both sham and coarcted animals. Blood pressure gradient difference between carotid and femoral blood pressures) was significantly greater in coarcted mice. Mean (**C**), systolic (**D**), and diastolic (**E**) blood pressures were higher in the carotid arteries of coarcted mice. The data are presented as the mean ± *SEM*, *n* = 6 to 10 per surgical group, * *p* <.05.

**Figure 5:**
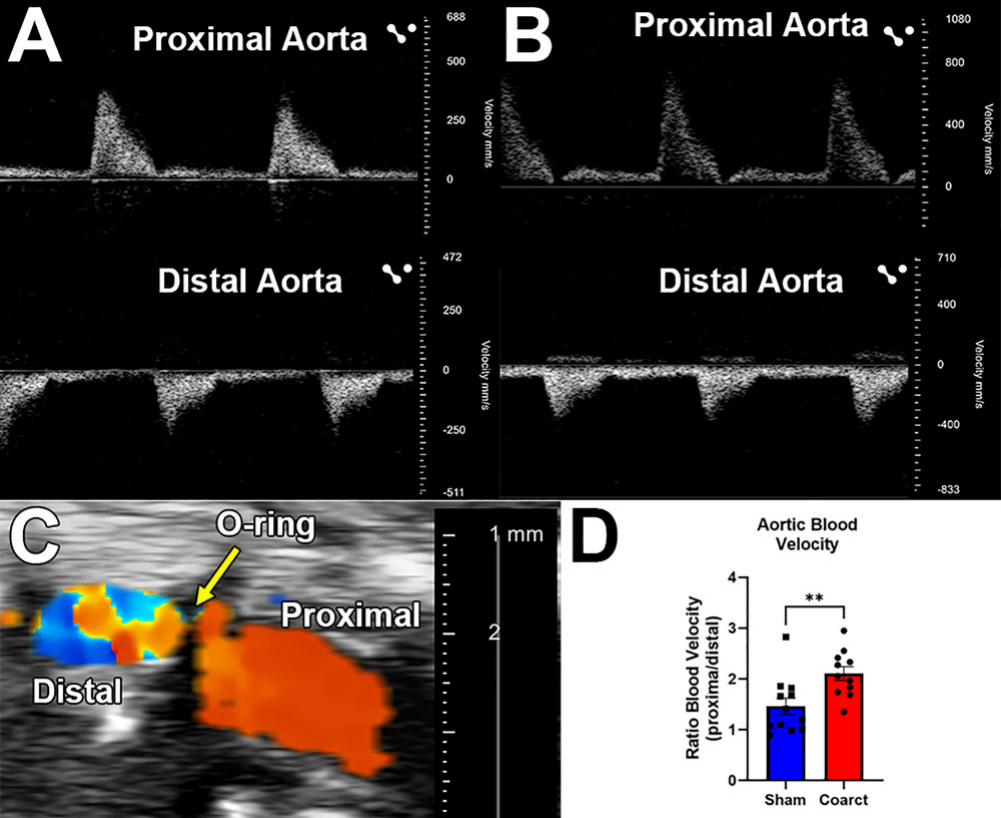
Doppler ultrasound images of the descending thoracic aorta from (**A**) sham and (**B**) coarcted mice. Blood velocity measurements were collected in both proximal (top) and distal (bottom) segments of the aorta. (**C**) Color flow Doppler imaging of the region of the aorta nearest the coarctation showing turbulent blood flow distal to the O‐ring (indicated by yellow arrow). (**D**) Average aortic blood velocity ratio (proximal/distal) in sham and coarcted mice. The data are presented as the mean ± *SEM*, *n* = 9 to 10 per surgical group.

If protocols are executed correctly, users should observe the following:

Physiological parameters: Heart rate and pulse pressure should remain within normal ranges across all groups if surgical recovery is adequate. Changes in heart weight/body weight ratio may indicate cardiac remodeling in response to altered hemodynamics (Fig. 3).
Pressure gradients (Basic Protocol 1 and 2): In coarcted animals, mean arterial pressure in the carotid artery should exceed femoral values by 10 to 15 mmHg. Sham animals should show negligible gradients (<2 mmHg). These gradients reflect the degree of aortic narrowing and are stable over the 8‐week observation period (Fig. 4).
Velocity ratios (Basic Protocol 3): Doppler ultrasound should reveal a ≥2‐fold increase in peak systolic velocity upstream vs downstream of the coarctation in affected animals. Sham‐operated mice typically have a ratio near 1.2 (Fig. 5).

Negative results, such as absent pressure gradients or velocity changes, typically reflect technical error during surgery or imaging and warrant method reassessment.

### Time Considerations

Basic Protocol 1 (surgical coarctation) takes 30 to 40 min per mouse, including anesthesia, ventilation, and closure. Basic Protocol 2 (telemetry implantation) takes 30 to 40 min per mouse. Basic Protocol 3 (Doppler ultrasound imaging) takes 30 to 40 min per animal per imaging session.

Complete experimental timelines may extend to ≥8 weeks, depending on longitudinal endpoints.

### Author Contributions

**Adam Lauver**: Conceptualization; data curation; formal analysis; funding acquisition; investigation; methodology; project administration; resources; software; supervision; validation; visualization; writing—original draft; writing—review and editing. **Hannah Garver**: Conceptualization; data curation; investigation; methodology; validation; writing—original draft; writing—review and editing. **Teresa Kreiger‐Burke**: Conceptualization; data curation; investigation; methodology; validation; visualization; writing—original draft. **Javier Rendón**: Data curation; formal analysis; investigation; methodology; visualization; writing—original draft. **Andres Contreras**: Data curation; formal analysis; funding acquisition; methodology; supervision; writing—original draft. **Stephanie Watts**: Conceptualization; funding acquisition; supervision; writing—original draft. **Gregory Fink**: Conceptualization; formal analysis; funding acquisition; methodology; supervision; writing—original draft.

### Conflict of Interest

The authors declare no conflicts of interest.

## Data Availability

The data, tools, and materials (or their source) that support the protocols are available from the corresponding author upon reasonable request.
